# Evolution of multiple phosphodiesterase isoforms in stickleback involved in cAMP signal transduction pathway

**DOI:** 10.1186/1752-0509-3-23

**Published:** 2009-02-20

**Authors:** Yukuto Sato, Yasuyuki Hashiguchi, Mutsumi Nishida

**Affiliations:** 1 Division of Molecular Marine Biology, Ocean Research Institute, The University of Tokyo, 1-15-1 Minamidai, Nakano-ku, Tokyo 164-8639, Japan

## Abstract

**Background:**

Duplicate genes are considered to have evolved through the partitioning of ancestral functions among duplicates (subfunctionalization) and/or the acquisition of novel functions from a beneficial mutation (neofunctionalization). Additionally, an increase in gene dosage resulting from duplication may also confer an advantageous effect, as has been suggested for histone, tRNA, and rRNA genes. Currently, there is little understanding of the effect of increased gene dosage on subcellular networks like signal transduction pathways. Addressing this issue may provide further insights into the evolution by gene duplication.

**Results:**

We analyzed the evolution of multiple stickleback phosphodiesterase (PDE, EC: 3.1.4.17) 1C genes involved in the cyclic nucleotide signaling pathway. Stickleback has 8–9 copies of this gene, whereas only one or two loci exist in other model vertebrates. Our phylogenetic and synteny analyses suggested that the multiple PDE1C genes in stickleback were generated by repeated duplications of >100-kbp chromosome segments. Sequence evolution analysis did not provide strong evidence for neofunctionalization in the coding sequences of stickleback PDE1C isoforms. On the other hand, gene expression analysis suggested that the derived isoforms acquired expression in new organs, implying their neofunctionalization in terms of expression patterns. In addition, at least seven isoforms of the stickleback PDE1C were co-expressed with olfactory-type G-proteins in the nose, suggesting that PDE1C dosage is increased in the stickleback olfactory transduction (OT) pathway. *In silico *simulations of OT implied that the increased PDE1C dosage extends the longevity of the depolarization signals of the olfactory receptor neuron.

**Conclusion:**

The predicted effect of the increase in PDE1C products on the OT pathway may play an important role in stickleback behavior and ecology. However, this possibility should be empirically examined. Our analyses imply that an increase in gene product sometimes has a significant, yet unexpected, effect on the functions of subcellular networks.

## Background

Duplicate genes generally persist and evolve through the partitioning of ancestral functions among the duplicates (subfunctionalization [1]) or the acquisition of novel functions through the fixation of beneficial mutations (neofunctionalization [2,3]). To date, many duplicate genes have been shown to have evolved through sub-/neo-functionalization in terms of the spatiotemporal pattern of their expression and/or the functional repertoire of their coding proteins [4-7]. Additionally, duplication may result in an increase in gene dosage that sometimes has advantageous effects, resulting in the maintenance of the duplicated genes [8]. For example, translational RNAs such as tRNA and rRNA, and structural proteins such as histones are often encoded by multiple gene copies [9-12]. This likely corresponds to the high demand of their gene products needed for translational and structural roles. Regarding subcellular networks, on the other hand, the genes involved in transcription regulations and signal transduction pathways were found to be over-retained in duplicate after whole genome duplication (WGD) in higher eukaryotes [13,14]. These data have been interpreted and discussed in the theoretical context of an increase of gene dosage [2,15-17]. However, it remains largely unexplored for possible effect of increased dosage of respective genes on overall function of subcellular networks, such as signal transduction pathways. These types of investigations may provide a more comprehensive understanding of evolution by gene duplication.

In a previous study of vertebrate genes involved in olfactory transduction (OT), we found that the three-spined stickleback *Gasterosteus aculeatus *has multiple duplicates of the phosphodiesterase (PDE, EC: 3.1.4.17) 1C gene (Sato Y, Hashiguchi Y, Nishida M: Temporal pattern of loss/persistence of duplicate genes involved in long-term potentiation, taste/olfactory transduction, and tricarboxylic acid cycle after teleost-specific genome duplication, submitted). In that study, we performed comparative analyses among four teleost and three tetrapod genomes to search for duplicate genes derived from the teleost-specific third-round (3R)-WGD [18,19] by focusing on several kinds of signal transduction networks. Data mining and phylogenetic analyses showed that the PDE1C gene, which decomposes cAMP and thus has a key role in the negative feedback of the OT [20,21], underwent 6–7 duplications in stickleback ancestor after its split with pufferfish. Thus, at least stickleback (and maybe also other species related to sticklebacks) has multiple PDE1C genes, whereas other model vertebrates including medaka, *Xenopus*, and human have only one or two PDE1C genes. However, the mechanisms for the maintenance of these PDE1C duplicates are unknown. The OT system, in which the PDE1C is involved, is expected to play an important role in the evolution of the stickleback, which demonstrates interesting ecological behaviors such as anadromous migration, territorial behavior, nest building, and parental care of eggs [22,23]. Thus, it is of interest to understand whether the multiple PDE1Cs in stickleback have persisted through sub-/neo-functionalization or by the effects of increased gene dosage in the OT system.

In this study, to explore the functional and evolutionary significance of the highly duplicated PDE1C genes in the stickleback, we carried out a comprehensive evolutionary analysis. First, we investigated the gene phylogeny and conserved synteny of the duplicated PDE1C genes to elucidate the chromosome/genome-level events that have generated the multiple PDE1Cs of stickleback. Second, based on the evolutionary framework obtained from the above investigation, the functional diversification of expression in organs and protein-coding sequences of the duplicated PDE1C genes were examined by gene expression and molecular evolutionary analyses. Third, we estimated the number of PDE1C loci involved in the OT of stickleback by analyzing co-expression between the PDE1Cs and olfactory-type G-protein (G [olf]: the guanine nucleotide-binding protein subunit alpha olfactory type). According to the result of the co-expression analysis, finally, we attempted to address the effect of increased PDE1C dosage on the function of the OT using *in silico *pathway simulation. Our results implied that the evolutionary significance of the duplicated PDE1C genes in stickleback is in the diversification of expression patterns and an increase in gene dosage, rather than neofunctionalization of the coding sequences.

## Results

### Phylogeny and synteny among stickleback PDE1C genes

**Figure 1:**
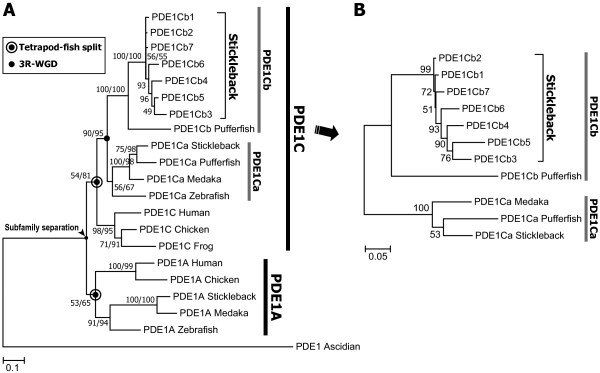
**Molecular phylogeny of vertebrate PDE1C**. **(A) **Maximum-likelihood (ML) tree of the PDE1C and PDE1A genes in four teleosts, three tetrapods, and an ascidian, constructed under the GTR + I + Γ model with 930 base pairs (bp) of the coding region. Numbers indicate support values (percentages) from 1,000 LR-ELW edge support tests (left) and percent posterior probabilities from the Bayesian method (right). Single numbers indicate the LR-ELW edge support for the nodes, for which the Bayesian tree inference resulted in a different branching pattern. **(B) **ML tree of the teleost PDE1C genes constructed under the TrN + Γ model with 1248 bp of the coding region. Numbers indicate the LR-ELW edge support values (1,000 replications).

The maximum likelihood (ML)/Bayesian molecular phylogeny of chordate PDE1A and PDE1C (Figure 1) showed that seven of the duplicated PDE1C genes in stickleback (PDE1Cb1-b7) arose from repeated duplications that occurred after the divergence between stickleback and pufferfish (see Figure 1B). In addition, the teleost (zebrafish, medaka, stickleback, and pufferfish) PDE1C genes were grouped within two major clades, PDE1Ca and PDE1Cb, with relatively high support values (see black circle in Figure 1A; LR-ELW [the expected-likelihood weights applied to local rearrangements of tree topology] [24] edge support = 90%; Bayesian posterior probability = 95%). This branching pattern suggests that the PDE1C gene was duplicated in a teleost ancestor after its split from the tetrapods (see double circle in Figure 1A), possibly through the 3R-WGD [18,19]. This possibility was examined by investigating the synteny of the surrounding genes of the PDE1C loci in teleosts and tetrapods as described below.

**Figure 2:**
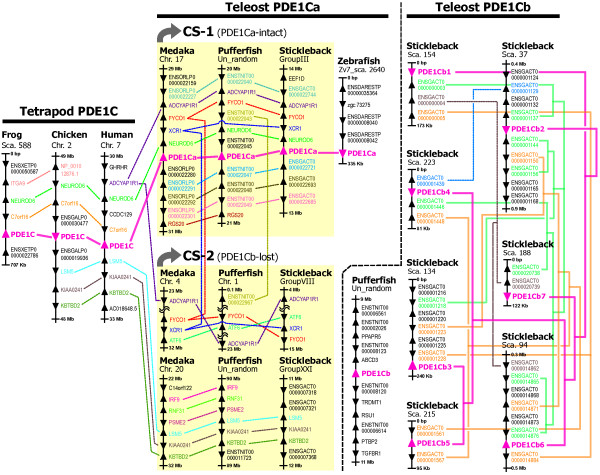
**Conserved synteny around the PDE1C locus (loci) in tetrapods and teleost fishes**. Triangles indicate gene loci and their direction of transcription. Doubly conserved synteny, which was derived from the teleost-specific genome duplication [18,19], is indicated by yellow shading and labeled "CS (conserved synteny)-1" and "CS-2." Orthologous/paralogous relationships among PDE1C genes are shown by solid magenta lines. The dashed lines indicate putative orthologous relationships predicted in the Ensembl genome database [40]. The solid yellow and green lines show phylogenetic relationships of neighboring "unknown" genes around stickleback PDE1Cb loci, which are estimated in the present study [see Additional file 1, Figure S1]. The PDE1Cx (Ensembl ID: ENSGACP00000001336) [see Additional file 1, Table S1] of the stickleback is located alone in a small contig (Scaffold 809; 12 kbp), and therefore has no synteny information.

To clarify the genomic events that generated the two paralogous genes in teleosts (PDE1Ca and PDE1Cb) and multiple PDE1Cb genes in stickleback, we investigated the genomic regions around the PDE1C loci. We found conserved synteny between the PDE1C locus in tetrapods (human, frog, and chicken) and the PDE1Ca locus in medaka, stickleback, and pufferfish (described as "conserved synteny [CS]-1," Figure 2). One more homologous region corresponding to the CS-1 was found in medaka, pufferfish, and stickleback (described as "CS-2," Figure 2). CS-1 and CS-2 were considered doubly conserved syntenies derived from the 3R-WGD, which corresponds to a single tetrapod chromosome segment. However, CS-2 appeared to have lost the putative region that contains PDE1Cb. Zebrafish PDE1Ca, pufferfish PDE1Cb, and stickleback PDE1Cb1-b7 were found within other chromosome segments, implying secondary translocation(s) of these PDE1C genes after the 3R-WGD. The pufferfish PDE1Cb region partially corresponded to human chromosomes 1 (94.6–99.6 Mb) and 10 (16.6–17.3 Mb) [see Additional file 1: Table S1]. However, we were unable to find human chromosome regions corresponding to the zebrafish PDE1Ca and stickleback PDE1Cb1-b7 regions. Among the stickleback PDE1Cb1-b7 regions, the surrounding genes were very similar (see "Teleost PDE1Cb" in Figure 2), implying that repeated segmental duplications generated the multiple PDE1Cb regions in stickleback. We found one more ninth PDE1C locus in stickleback (provisionally named PDE1Cbx) [see Additional file 1: Table S1], whose sequence was 5'-and 3'-truncated and partial (246 residues in total whereas the others have >395 residues) probably due to incompleteness of the sequence contig where the locus was located (scaffold_809: only 11,237 base pairs [bp] in total). We could not conclude whether this is pseudogene or not, and thus we did not include this locus in our analysis.

Our overall results from the phylogenetic and synteny analyses clearly revealed the evolutionary relationships among PDE1C genes in the bony vertebrates and the evolutionary origin of multiple PDE1C genes in stickleback. This provided the basis for our subsequent analysis on the molecular evolution of the multiple stickleback PDE1C genes.

### Molecular evolution of multiple PDE1C genes

**Figure 3:**
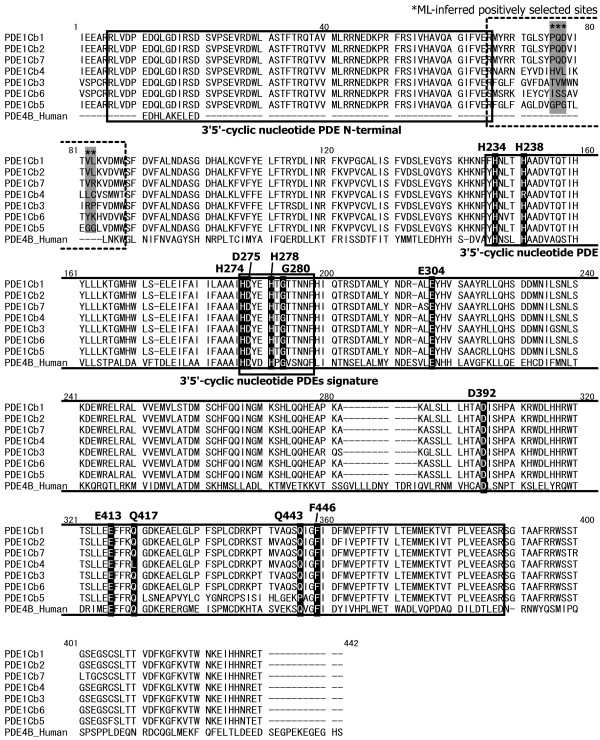
**Protein sequence alignment of multiple stickleback PDE1Cb and human PDE4B**. The PDE tertiary structure and active enzyme sites (black shading) are reported for the human PDE4B (site numbers of active sites are according to ref. [28]). The signature domains of PDE protein are designated by solid boxes, and the sequence region that contains the ML-inferred positively-selected sites is designated by a dashed box. The stars above and gray shading indicate the inferred positively selected sites.

The multiple PDE1C genes of stickleback were analyzed by ML-estimation of the nonsynonymous to synonymous substitution rates (*d*_N_/*d*_S _= ω) during evolution, which is a possible indicator of adaptation at the protein sequence level [25-27]. In this analysis, we tested whether some portion of the PDE1Cb sequences shows ω >1, which is the signature of adaptive amino acid changes, by comparing maximum-likelihood values of simple evolution model having fewer ω parameter(s) (M0, M1, and M7 in Table 1) with those of more complex model having more ω parameters, some of which were allowed to be >1 (M2, M3, and M8 in Table 1; for details, see Methods). The likelihood ratio test (LRT)-1, the comparison between M0 with M3, implied that the stickleback PDE1Cb genes (PDE1Cb1-b7) were under positive diversifying selection with regard to their protein sequences (ω_2 _= 17.83). However, the LRT-2, LRT-3, and "branch-site test", the comparison between M1 and M2, M7 and M8, and M2 and M3, respectively, did not support this implication (ω_2 _= 0.15, ω_2 _= 0.45, and ω_2 _= 0.08, respectively; see Table 1). The LRT-1 (M3 model) detected five codon sites under positive selection, including sites 76–78, 82, and 83 (indicated by stars in Figure 3). On the other hand, the LRT-2, LRT-3, and branch-site test (ω >1) did not identify these individual sites as being under positive selection.

**Table 1:** Log-likelihood scores (*l*) and parameter estimates under several models for ω estimates in multiple PDE1Cb genes of stickleback

Models	*l*^a^	Average ω^b^	ML-estimated parameters
*Site-specific model*			
LRT-1			
One ω ratio model (M0)	-5866.90	0.1395	*P*_0 _= 1.0000, ω_0 _= 0.1395
Discrete model (M3)	-5723.25	0.3937	*P*_0 _= 0.7798, *P*_1 _= 0.2063, *P*_2 _= 0.0139, ω_0 _= 0.0543, ω_1 _= 0.5031, ω_2 _= 17.8270
-2Δ*l*_ [d.f. = 4]_	287.29 *		
LRT-2			
Neutral model (M1)	-5906.08	0.4735	*P*_0 _= 0.5265, *P*_1 _= 0.4735, ω_0 _= 0.0000, ω_1 _= 1.0000
Selection model (M2)	-5743.66	0.1743	*P*_0 _= 0.3524, *P*_1 _= 0.0911, *P*_2 _= 0.5565, ω_0 _= 0.0000, ω_1 _= 1.0000, ω_2 _= 0.1495
-2Δ*l*_ [d.f. = 2]_	324.84 *		
LRT-3			
Beta model (M7)	-5763.28	0.1814	*p *= 0.3278, *q *= 1.3121, *P*_0 _= *P*_1 _= *P*_2 _= 0.3333, ω_0 _= 0.0030, ω_1 _= 0.0866, ω_2 _= 0.4548
Beta and ω model (M8)	-5763.28	0.1814	*p *= 0.3278, *q *= 1.3121, *P*_0 _= *P*_1 _= *P*_2 _= 0.3333, *P*_3 _= 0.0000, ω_0 _= 0.0028, ω_1 _= 0.0866, ω_2 _= 0.4548, ω_3 _= 0.3544
-2Δ*l*_[d.f. = 2]_	0.00		
*Branch-site model*			
Selection model (M2)	-5833.54	N.A.	*P*_0 _= 0.0939, *P*_1 _= 0.0652, *P*_2 _= 0.8409, ω_0 _= 0.0000, ω_1 _= 1.0000, ω_2 _= 0.0695
Discrete model (M3)	-5741.70	N.A.	*P*_0 _= 0.3084, *P*_1 _= 0.0562, *P*_2 _= 0.6354, ω_0 _= 0.0634, ω_1 _= 0.8749, ω_2 _= 0.0796

^a ^Log-likelihood scores.

^b ^Nonsynonymous–synonymous substitution ratio (ω = *d*_N_/*d*_S_) averaged over sites.

* *p *< 0.001

The positively selected sites inferred by the LRT-1 described above were not located on the known active sites of the enzyme or on specific domains or motifs of the PDE proteins (Figure 3). The active enzyme sites that are generally conserved in PDE proteins (indicated by black shading in Figure 3) [28] were also conserved among the stickleback PDE1Cb genes, with exceptions at sites 238 in PDE1Cb4, 417 in PDE1Cb4, and 443 in PDE1Cb5. Moreover, the known PDE-specific protein domains were successfully detected in all stickleback PDE1Cb genes (indicated as solid boxes in Figure 3) via queries to protein domain databases Pfam [29] and PROSITE [30], suggesting that they conserve PDE function. On the other hand, the sequence region containing the inferred positively-selected sites (sites 66–88; indicated as dashed boxes in Figure 3) yielded no hits in these databases. Those positively-selected sites were distributed on the opposite side of a substrate-binding pocket of this enzyme [see Additional file 1: Figure S2]. In addition, this region did not correspond to the important variant region of the three functional splicing variants reported in mouse PDE1C [21].

### Spatial expression of the multiple PDE1C genes

**Figure 4:**
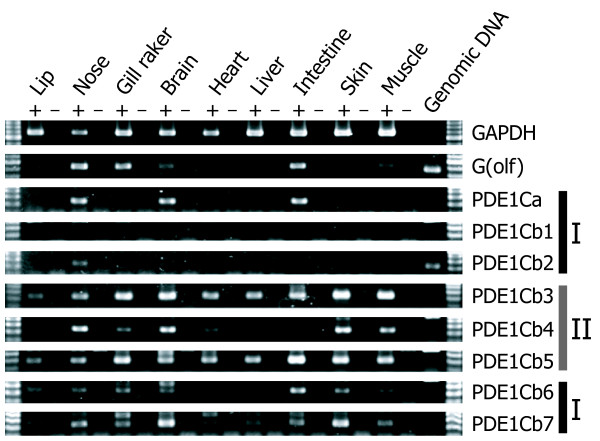
**Spatial expression patterns of PDE1C and G(olf) in the stickleback**. Semi-quantitative RT-PCR analysis was performed to assess expression levels and patterns of G-protein subunit alpha olfactory type (G [olf]) and multiple PDE1C genes in stickleback. Plus (+) and minus (-) signs indicate PCR assays using reverse-transcribed cDNA for each tissue type and assays using total RNA without reverse transcription (negative controls), respectively. The glyceraldehyde-3-phosphate dehydrogenase (GAPDH) gene was amplified as a positive control. The overall expression patterns of the genes were essentially similar between the two stickleback individuals investigated.

Spatial expression patterns across tissues were investigated and compared among the multiple PDE1C and G(olf) genes in adult stickleback (Figure 4). Reverse transcription polymerase chain reaction (RT-PCR) analysis showed that seven of the eight PDE1C genes were co-expressed with G(olf) in the nose of stickleback. Among these seven genes, five (PDE1Ca, PDE1Cb3, b4, b5, and b7) were strongly expressed, and two (PDE1Cb2 and b6) were weakly expressed in the nose. In addition, those stickleback PDE1Cs were roughly divided into two groups in terms of their overall expression patterns. One group included the PDE1Cs that were expressed strongly in a few tissues ("group I"; 0–3 tissues; PDE1Ca, PDE1Cb1, b2, b6, and b7) and the other group included the PDE1Cs that were strongly expressed in several tissues ("group II"; 4–9 tissues; PDE1Cb3, b4, and b5). Among the "group I" genes, the expression of PDE1Ca was clearly detected in the nose, brain, and intestine and was similar to that of G(olf), which was strongly expressed in the nose, gill raker, and intestine. On the other hand, PDE1Cb1, b2, b6, and b7 seemed to have lost the strong co-expression with G(olf) in the nose (PDE1Cb6), intestine (PDE1Cb7), or both tissues (PDE1Cb1 and b2). Among the "group II" genes, PDE1Cb4 was strongly expressed in the nose, brain, skin, and skeletal muscle, and PDE1Cb3 and b5 were strongly expressed in almost all tissues.

### *In silico *simulations for the multiple PDE1C genes

**Figure 5:**
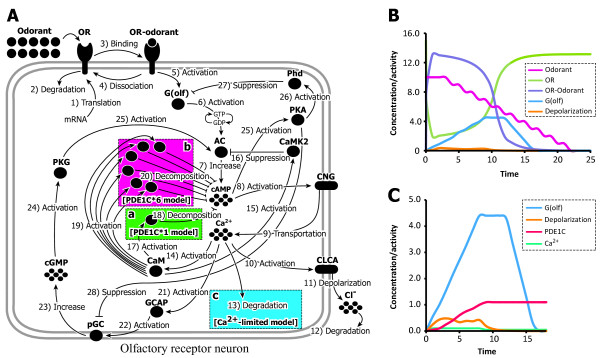
**Schematic view of a simulation model of the olfactory transduction (OT) pathway and its simulation performance**. **(A) **OT simulation model constructed under the KEGG pathway database [31]. **(B) **Observed resultant oscillations of the odorant, OR, OR-odorant complex, G(olf), and depolarization under the "single-PDE1C [threshold = 1]" model. The x-axis indicates the simulation time scale, and the y-axis indicates the concentration of the odorant and/or activity intensities of involved proteins. **(C) **Observed oscillations of the G(olf), depolarization, Ca^2+^, and PDE1C, the latter two are the key molecules of the negative feedback circuit of the OT, which finally blocks the depolarization.

We examined the possible effects of increased gene/product dosage from the multiple PDE1C genes in stickleback on the output (depolarization) of the OT pathway using *in silico *simulations. Figure 5 depicts a schematic diagram of the OT simulation model constructed according to the KEGG pathway database (panel A) [31], and a representative result of the simulation (panels B and C; the case of "single-PDE1C [threshold = 1]" model). Figure 5B shows that the odorant and olfactory receptor (OR) produced OR-odorant complex (violet line) that stimulated the G(olf) (blue line), led to depolarization occurring on the simulation time scale of 0–10 (orange line). The depolarization is blocked by activation of the PDE1C. Figure 5C shows that the activated G(olf) increased the concentration of Ca^2+ ^(green line), which stimulates PDE1C (red line) via the mediation of calmodulin (CaM; see Figure 5A). When the activity level of the PDE1C has gone over its firing threshold (set to 1 here; see Figure 5C), the activated PDE1C decomposed cAMP (see Figure 5A), and finally terminated the depolarization (see Figure 5C). Although this preliminary model approach may be simple, we thought that this model would provide a basic framework to analyze the effect of an increased dosage of a specific element; that is, the PDE1C gene. We compared the depolarization signals of the "single-PDE1C" model and "multiple-PDE1Cs" model. Since five PDE1Cs were strongly expressed, and two were weakly expressed in the nose (see Figure 4), we set the number of PDE1C circuits equal to six in the "multiple-PDE1Cs" model as an approximate representation of the estimated gene dosage in the OT.

**Figure 6:**
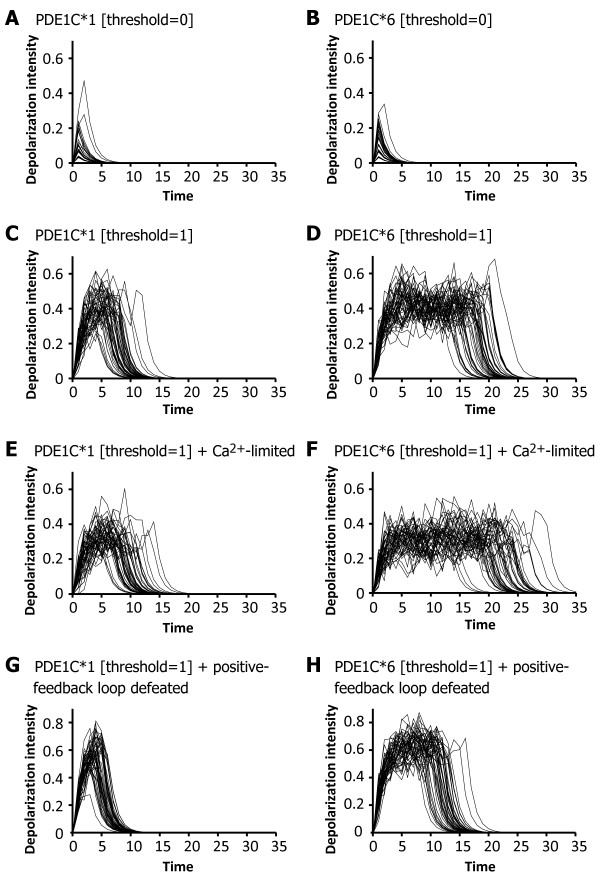
**Comparison of the simulated depolarization signals between single- and multiple-PDE1C models**. Simulation results of single- and multiple-PDE1C models are shown in the left and right panels, respectively. The x-axis indicates the simulation time scale, and the y-axis indicates the intensity of depolarization of the olfactory receptor neutron. The values of "threshold" indicate the firing threshold of PDE1C in terms of their activity levels (see Figure 5C) set in the respective simulations. Depolarization signals were obtained using 50 replications of the respective simulation. **(A) **Results under the single-PDE1C model in which the firing threshold of PDE1C was set to 0 (threshold = 0). **(B) **Results under the multiple-PDE1C model (threshold = 0). **(C) **Results under the single-PDE1C model (threshold = 1). **(D) **Results under the multiple-PDE1C model (threshold = 1). **(E) **Results under the model of single-PDE1C (threshold = 1) and limited Ca^2+ ^availability. **(F) **Results under the model of multiple-PDE1C (threshold = 1) and limited Ca^2+ ^availability. **(G) **Results under the model of single-PDE1C (threshold = 1) and positive feedback circuit knocked out. **(H) **Results under the model of multiple-PDE1C (threshold = 1) and positive feedback circuit knocked out.

Our OT model simulations implied that the increase in the number of PDE1C circuits affects the longevity of the depolarization signal (Figure 6). In the case of the single- and multiple-PDE1C models with a PDE1C threshold = 0, the resultant depolarization signals were weak (intensity = 0.05–0.2) and short-lived (longevity = 5) when the number of PDE1C circuits was one or six (Figure 6A and 6B). In these simulation models, PDE1C seems to work rapidly after odorant stimulation, leading to an instant block of depolarization by the PDE1C circuit regardless of whether multiple PDE1Cs were involved. When the PDE1C threshold = 1, on the other hand, the resultant depolarization signals became more intense (intensity = 0.3–0.5; Figure 6C and 6D), and the "multiple-PDE1C [threshold = 1]" model yielded an elongated signal of depolarization (longevity = 17–25; Figure 6D) compared to the "single-PDE1C [threshold = 1]" model (longevity = 8–14; Figure 6C).

We also found that the depolarization longevity of the "multiple-PDE1C [threshold = 1]" model was further extended by limiting the availability of Ca^2+^, which is an upstream regulator of the PDE1C circuit. Ca^2+ ^dosage limitation affected the single- and multiple-PDE1C [threshold = 1] models similarly in terms of their depolarization intensities, which were reduced to 0.2–0.4, compared to the model with unlimited Ca^2+ ^(Figure 6E and 6F). In the "multiple-PDE1C [threshold = 1] + Ca^2+^-limited" model, the longevity of the depolarization signal was greatly elongated (longevity = 25–30; Figure 6F), compared to the "single-PDE1C [threshold = 1] + Ca^2+^-limited" model (longevity = 10–15; Figure 6E).

Since the PDE1C is activated by Ca^2+^-activated CaM, it is possible that the finite Ca^2+ ^dosage invoked competition among increased PDE1Cs, resulting in a delay in blocking of the depolarization. This situation likely led to a positive-feedback circuit (the processes of 21–25 shown in Figure 5A), also activated by upstream Ca^2+^, to be more prevalent in the OT system. Therefore, the depolarization longevity was extended in the "multiple-PDE1C" models (see Figure 6D and 6F). This possibility was supported by an *in silico *mutation analysis (Figure 6G and 6H), in which we knocked out the positive feedback circuit (processes 21–25 and the related elements) in the simulation model. In this "multiple-PDE1C [threshold = 1] + positive-feedback loop defeated" model, the resultant depolarization signals were not greatly elongated (longevity = 13–17; Figure 6H), and the longevity was similar to that of the "single-PDE1C [threshold = 1]" model (Figure 6C).

## Discussion

### Evolutionary origins of the multiple PDE1C genes in stickleback

Molecular phylogenies (Figure 1) and conserved synteny (Figure 2) analyses showed that the teleost PDE1C was duplicated through the 3R-WGD, generating two isoforms, PDE1Ca and PDE1Cb, and that PDE1Cb was repeatedly duplicated in the stickleback lineage 6–7 times through chromosome segment duplications. Such multiple gene copies could be generated by fewer than six duplications especially in tandemly located genes; however, this seems not to be the case of the stickleback PDE1Cb genes, which are located on different scaffolds respectively. The stickleback PDE1Cb loci and the orthologous pufferfish PDE1Cb locus appears to have translocated from CS-2, which is one of the pair of conserved chromosome regions derived from 3R-WGD. The corresponding PDE1Cb locus in medaka and zebrafish has been lost (Figure 2). Since no synteny was found between stickleback PDE1Cb loci and the pufferfish PDE1Cb locus, the translocation of the PDE1Cb gene may have occurred independently in the two lineages. Alternatively, the PDE1Cb translocation may have occurred once in an earlier ancestor, and either the stickleback or pufferfish experienced an additional PDE1Cb translocation. After the translocation(s), the stickleback PDE1Cb gene along with the neighboring tandemly repeated "unknown" genes (denoted by Ensembl transcript IDs in Sca. 37, 94, 134, 154, 188, 215, and 223 in Figure 2) appeared to have undergone lineage-specific expansion (LSE) [32] by chromosome segment duplications of >100 kbp. Such plasticity in the evolution of the 3R-WGD-derived duplicates, such as an independent loss or persistence among teleost lineages, is also shown in a recent study on androgen receptors [33]. The persistence of these receptors in various Percomorph fishes is suggested to be responsible for their neofunctionalization in ligand-binding activities that may be associated with plasticity of sex determination in those teleosts. It is also interesting to explore how the multiple stickleback PDE1Cb loci presented here have been retained.

The various stickleback chromosome segments that contain PDE1Cb1-b7 (see Figure 2) may have been retained because of advantages related to the LSE of PDE1Cb and/or the "unknown" genes mentioned above. LSEs are thought to have played an important role in the proteome evolution of multicellular eukaryotes [32], particularly in the evolution of proteins involved in ligand recognition, pathogen resistance, etc [34-36]. The function of the "unknown" genes is not annotated in the Ensembl stickleback genome. However, at least a part of their corresponding human genes is suggested to function as an angiotensin II/arginine vasopressin (AII/AVP) receptor-like protein responsible for an autoimmune disease (cold autoinflammatory syndrome) (found by a BLASTP search; data not shown). The "unknown" stickleback genes may also have immune-related functions. The repeated segmental duplication of these "unknown" genes (see Figure 2) might be advantageous in stickleback responses to diverse pathogens or other ligands. This possibility should be examined further. On the other hand, the stickleback PDE1Cb genes are intact and do not have stop codons or frameshifts in their coding sequences. Thus, they should also have been allowed to be highly duplicated. Accordingly, the PDE1Cb genes in stickleback may also have important phenotypic consequences; for this reason, we analyzed their molecular evolution, gene expression, and possible gene dosage effects, as discussed below.

### Sequence and expression evolution of the multiple stickleback PDE1C genes

The sequence evolution analysis of the multiple PDE1Cb genes in stickleback did not provide strong evidence for neofunctionalization [1,2] in their coding proteins. The known active sites and specific domains/motifs of PDE enzymes were highly conserved among the multiple PDE1Cb genes (Figure 3). In addition, although the ML-estimation of the *d*_N_/*d*_S _ratio implied adaptive sequence evolution in the case of LRT-1 (Table 1), the estimated positively-selected amino acid sites are located on the opposite side of a substrate-binding pocket of the PDE1 [see Additional file 1: Figure S2] and these positively-selected sites are included in the sequence region for which the possible function is not reported (see Results). From these observations, we can hardly conclude that the multiple PDE1Cb genes were maintained through the acquisition of new enzyme functions. This implies that evolutionary mechanisms other than neofunctionalization in coding sequence may have retained the multiple stickleback PDE1C genes.

Gene expression analysis (Figure 4) showed that the PDE1Ca and relatively "basal" PDE1Cb genes (see Figure 1B; designated as "group I" in Figure 4) were strongly expressed in a few (0–3) tissues, and their expression patterns were similar to G(olf). Since PDE1C and G(olf) are involved in the OT [20,21,37], expression patterns of these basal ("group I") PDE1Cb genes seem to represent ancestral states of the teleost PDE1Cb. This might be verified by further expression analysis on the PDE1Cb from many other teleosts in the future. On the other hand, the "derived" PDE1Cb genes (designated as "group II" in Figure 4) were strongly expressed in many more tissues, implying that they acquired expression in new organs. These PDE1Cb genes, which seem to be neofunctionalized in terms of expression patterns, may be under functional adaptation to their new subcellular environments. Furthermore, they might experience neofunctionalization through the acquisition of new mutations in the future.

In addition, the number of PDE1C loci involved in the stickleback OT seems to be increased, as suggested by the fact that at least seven PDE1C isoforms were co-expressed with G(olf) in the stickleback nose (Figure 4). Such an increase in PDE1C gene products may have some functional significance in the performance of the OT. However, directly addressing this question is difficult within the scope of this study, because it would require gene knockout, neurophysiological, and behavioral analyses. Furthermore, gene expression levels of the PDE1C would be assessed more quantitatively by a real-time PCR approach. Such an approach will also useful to explore the existence of gene number variation of PDE1C within and between populations of stickleback, which includes a variety of ecomorphs (e.g., marine, anadromous, and freshwater populations) [22,23] in the future. To obtain and describe the primary predictions of the phenotypic effects from increased PDE1C dosage, we used *in silico *pathway simulations, as discussed below.

### Possible effect of increased PDE1C products on olfactory transduction

The effect of increased PDE1C dosage on stickleback OT was surveyed using *in silico *pathway simulation. The simulation was based on limited information and knowledge of OT, and the predictions resulting from the simulation should be empirically evaluated. Regardless, such approaches may provide insight into the evolutionary significance of the multiple PDE1C genes in the stickleback.

According to the results of the OT simulation, the increased PDE1C dosage extends the longevity of the depolarization signal of the olfactory receptor neuron (Figure 6). PDE1C is involved in the negative feedback circuit of the OT and decomposes cyclic adenylic acid (cAMP) and eventually terminates the depolarization depending on upstream Ca^2+^/CaM [20,21,38] (see Figure 5). Accordingly, the increased PDE1C products may compete with each other for binding to these upstream Ca^2+^/CaM molecules, and consequently delay the termination of depolarization (Figure 6C and 6D). This proposed delay mechanism seems to be supported from our additional simulation results that Ca^2+^/CaM limitation further extends the longevity of the depolarization signal (Figure 6E and 6F). Competition among the increased PDE1Cs involved in the negative feedback circuit may result in positive feedback in the OT system to be dominant. This situation would also elongate the depolarization signal. Our *in silico *mutation-simulation analysis supported this hypothesis (Figure 6G and 6H). That is, a defect in the positive feedback circuit antagonized the effect of the increased PDE1C dosage, suppressing the depolarization elongation. To summarize, our simulation analyses suggested that increased PDE1C dosage induces competition among PDE1Cs and causes the positive feedback loop to be dominant in the OT system, resulting in a delay in the termination of the output depolarization of the olfactory receptor neuron.

It is proposed that an extension in the duration of olfactory signals is associated with the territorial ecology of the house mouse *Mus domesticus *[39]. In *M. domesticus*, the male scent mark contains lipocalin proteins called major urinary proteins (MUPs). The MUPs bind with the semiochemical molecules and release them gradually, which eventually extends the longevity of the odor signal. This makes it difficult for other male mice to tell whether the odor signals come from scent marks or a territorial male, and the other males are hesitant to approach the territorial zone because the scent-mark odor has an aggressive message. This is thought to be evolutionarily advantageous for both the territorial and the other individual because potential male invaders reduce their risk of damage or death due to conflict [39]. Although this phenomenon and the underlying mechanisms in house mice are different from those proposed for the stickleback, the multiple PDE1C genes may play an important role in stickleback ecology and behavior. This speculation may be appealing when considering that sticklebacks also hold territories where they build nests and reproduce.

Of course, these hypotheses should be empirically examined. Additionally, the application of the vertebrate OT system described in the KEGG [31] to this stickleback study should be verified in future research. However, we propose that the evolutionary significance of multiple gene duplicates may be evaluated more comprehensively using available biological information and analytical tools such as whole genome sequences, pathway data, and *in silico *simulation software, as was attempted here. Such a comprehensive approach would be particularly favorable for questions that are not entirely addressed using the molecular evolutionary analysis of a particular gene/protein. For example, this approach could be used in testing the effects of increased gene dosage in signal transduction pathways. With improvements in the pathway models and their parameters, the *in silico *pathway simulation, which can perform a synthetic analysis of molecular dynamics for multiple gene products and other biomolecules, will become one of the most powerful approaches in understanding complex macro-phenotypic evolution.

## Conclusion

In this paper, we presented the results of a comprehensive analysis of the evolution of multiple PDE1C genes in the stickleback involved in a cAMP-mediated signal transduction pathway. Our results suggested that the PDE1C genes are evolutionary significant through either their diversification in expression among organs and/or through an increased gene dosage effect on the olfactory transduction pathway, rather than through neofunctionalization of their coding sequences. In particular, *in silico *simulation analysis implied that an increase of PDE1C dosage extends the longevity of olfactory signals. An increase in gene product may have a substantial effect on the functions of subcellular networks.

## Methods

### Phylogenetic analysis

To analyze the evolutionary origins and relationships of the multiple PDE1C genes in the stickleback, we performed a phylogenetic analysis of PDE1C and its closely related PDE1A genes from eight chordate species (human, chicken, frog, pufferfish, medaka, stickleback, zebrafish, and ascidian) using available whole-genome sequence data. The primary sequences of the PDE1 genes were gathered via queries to the Ensembl genome database [40] and its Ortholog Predictions section. We confirmed that no additional PDE1A and PDE1C genes existed in these genomic sequences using BLAST searches (*E*-value cut-off of < 10^-3^). When a partial sequence was detected in the Ensembl database, we predicted the full-length coding sequence from the genomic sequence using WISE2 [41]. The corresponding PDE1 of the sea lamprey *Petromyzon marinus *was searched using BLAST against the UCSC Genome Browser Database [42]. However, we were unable to find the full length sequence, which can be used as an outgroup for the phylogenetic analysis. The species names and Ensembl IDs of the analyzed PDE1A and PDE1C genes are provided in supplementary table S2 [see Additional file 1].

The nucleotide sequences of the PDE1A and PDE1C genes from the seven vertebrates and ascidian (outgroup) were aligned using ClustalW [43]. The alignment was manually adjusted according to the amino acid sequences using MacClade ver. 4.06 [44]. After removing the gaps, 930 bp of the PDE1 coding region were phylogenetically analyzed using maximum likelihood (ML) and Bayesian methods in TREEFINDER (version June, 2007) [45,46] and MrBayes (version 3.0b4) [47] under the GTR + I + Γ model [48], which was selected as the best-fitting model for nucleotide substitution by hierarchical LRT (hLRT) [49]. The ML analysis was assessed using 1000 replications of the LR-ELW edge support tests [24]. Bayesian posterior probabilities of the phylogeny and its branches were determined from 9901 trees. The re-aligned teleost PDE1C genes (1248 bp), excluding the partial zebrafish PDE1Ca (1185 bp), were analyzed using the ML method under the TrN + Γ model of nucleotide substitution [50], which was chosen by the hLRT. Bayesian method was not applied to this analysis, because MrBayes does not allow to use the TrN model.

### Synteny analysis

To investigate the chromosomal/genomic events that generated multiple PDE1C genes in the stickleback, genomic regions around the stickleback PDE1C loci were investigated and compared to those of human, chicken, frog, pufferfish, medaka, and zebrafish. Physical mapping data nearby each PDE1C locus were obtained from the Ensembl database [40]. An orthology of the neighboring genes [see Additional file 1: Table S1] within each species was examined according to descriptions in the Orthologous Prediction section of Ensembl database. Phylogenetic relationships of a part of neighboring genes of the stickleback PDE1Cb loci were analyzed by ML method according to the procedure described above [see Additional file 1: Figure S1]. The genomic location data of the genes near the PDE1C genes were used to rebuild the synteny maps.

### Molecular evolutionary analysis

To examine whether the multiple PDE1C genes in the stickleback were subjected to diversifying selection in terms of their amino acid sequences, we analyzed the *d*_N_/*d*_S _ratio (ω) using ML inferences of the ω values in codeml [25]. The re-aligned teleost PDE1C genes (1248 bp; 416 codons) were analyzed, excluding the partial zebrafish PDE1Ca (1185 bp) gene. An ML tree of these teleost PDE1C genes (shown in Figure 1B) was used as a reference tree. In this analysis, a simple ML model was compared to a more complex model having more parameters to obtain an adequate ω estimate. Statistical significance of the comparisons was assessed by LRT with degrees of freedom equal to the differences in the number of free parameters between the two models. First, the one-ratio model (M0), which assumes a one ω (ω_0_:0 < ω_0_<1) for all codon sites, was compared to the discrete model (M3), which assumes three ω (ω_0_, ω_1_, and ω_2_) for three site classes with proportions *p*_0_, *p*_1_, and *p*_2_. Second, the neutral model (M1), which assumes conserved sites (0<ω_0_<1) and neutral sites (ω_1 _= 1) with proportions *p*_0 _and *p*_1 _(*p*_1 _= 1-*p*_0_) was compared to the selection model (M2), which assumes the conserved, neutral, and selection sites with proportions *p*_0_, *p*_1_, and *p*_2_. The ω value of the selection sites (ω_2_) was allowed to be greater than 1. Third, the beta model (M7), which assumes that the ω varies according to the beta distribution β (*p*, *q*), was compared to the beta and ω model (M8), which assumes an additional site class with a ω value >1.

In addition, we performed a "branch-site test" [26] to detect positive selection at individual codon sites, if it exists, along respective branches leading to the multiple PDE1C genes of the stickleback. For this purpose, we set branches connecting the multiple PDE1C genes of the stickleback as "foreground" branches. The other branches leading to the pufferfish and medaka PDE1C genes were considered "background" branches. To obtain the adequate proportion estimates of site classes and their ω values, the selection model (M2) and discrete model (M3) were compared on the basis of their log-likelihood scores (*l*) estimated using codeml. Individual codon sites were assessed in terms of their posterior probability to belong to the site class for which the ω value was allowed to be >1.

### RT-PCR based co-expression analysis

To examine whether the multiple PDE1C genes in stickleback were involved in olfactory transduction (OT), we investigated the co-expression of the PDE1C genes and the G(olf) using semi-quantitative reverse transcription (RT)-PCR analysis. The gene-specific primers (GSPs) designed and used are described in Table 2. To distinguish the multiple PDE1C loci in stickleback, the 3' region of at least one primer from each primer pair was made to locate the differential nucleotide site among the PDE1C genes. For amplification of the G(olf) cDNA, a GSP pair was designed according to the nucleotide sequences of the stickleback G(olf) described in Ensembl (Ensembl Gene ID: ENSGACG00000016605 and ENSGACG00000001155).

**Table 2:** Gene-specific primers for RT-PCR-based expression analysis of PDE1C and G(olf)

Target gene	Sequence (5' → 3')^a^	Product length (base pairs)
Stickleback PDE1Ca	Forward: ATGGTGCATTGGTTGACTGA	233
	Reverse: CTCCAGTCGTCCTTGGAGAG	
Stickleback PDE1Cb1	Forward: CAAGGGCTTCAAGGTCACAT	152
	Reverse: CCTTTTCCTCCAGGTCTTCC	
Stickleback PDE1Cb2	Forward: ACAGACGGACCTCCAACATC	238
	Reverse: TGTTTGCTGTAGCCCACTTG	
Stickleback PDE1Cb3	Forward: CAAAGGCTTGTCCCTGCTAC	217
	Reverse: TGGTTCCACCATGAAGTCAA	
Stickleback PDE1Cb4	Forward: TGGGCTACAGCAAACACAAG	243
	Reverse: AGTTCTCCAAAGCTCGGTCA	
Stickleback PDE1Cb5	Forward: CACTGGCTCAGTGAGTTGGA	185
	Reverse: CTGTGCTGTAGAAGGCGACA	
Stickleback PDE1Cb6	Forward: CACTGGCTCAGTGAGTTGGA	239
	Reverse: AGCTCTCTGCTCCACTCGTC	
Stickleback PDE1Cb7	Forward: CTCCTTGGAAGTGGGCTACA	230
	Reverse: TGTACAACATGGCGGTGTCT	
Stickleback G(olf)	Forward: SAGCAGCAGCTACAACATGG	411
	Reverse: CATTCKCTGGATGATGTCMC	

^a ^Positions with mixed bases are designated by their IUB (the International Union of Biochemistry) codes: R = A/G; Y = C/T; K = G/T; M = A/C; S = G/C; W = A/T.

For the RT-PCR experiment, we used two adults of the anadromous form of the three-spine stickleback. Live specimens were collected at Akkeshi Lake, Hokkaido, Japan, in May 2008, and were treated according to the ethical recommendations of the Ichthyological Society of Japan and the University of Tokyo. Total RNA was extracted from the lip, nose, gill raker, brain, heart, liver, intestine, skin, and skeletal muscle of fresh stickleback samples, using 1 ml TRIZOL reagent (Invitrogen). Residual genomic DNA was removed using DNase I (Takara), and 168 ng of the repurified total RNA from each tissue were reverse-transcribed into first-strand cDNA with oligo-dT adaptor primer using TaKaRa RNA PCR kit ver. 3.0 (Takara). Genomic DNA was also extracted from a piece approximately 5 mg in size of the caudal fins using the AquaPure DNA extraction kit (BioRad).

To assess the expression patterns of the PDE1C and G(olf) genes across tissues, reverse-transcribed cDNA from each tissue was subjected to PCR reactions with the GSPs (Table 2). The thermal-cycle profile was as follows: 1 cycle at 94°C for 2 min; 35 cycles at 94°C for 30 sec, 55°C for 30 sec, and 72°C for 30 sec; followed by 1 cycle at 72°C for 3 min. As a positive control for gene expression, glyceraldehyde-3-phosphate dehydrogenase (GAPDH) was amplified using a primer pair designed by Aoki et al. [51]. As a negative control, PCR amplification was also conducted for each RNA sample without a reverse-transcribed reaction. The amplified DNA fragments were separated by electrophoresis on a 2% LO3 agarose gel (TaKaRa; 35 min at 50 V; constant voltage setting), stained with ethidium bromide, and visualized under UV light. GeneRuler 100-bp DNA Ladder Plus (MBI Fermentas) was used as a size marker.

### Pathway simulation

We examined the possible effect of the multiple PDE1C genes on the output (depolarization) of the OT system using *in silico *pathway simulation. As the modeling framework for the simulation, we chose Hybrid Functional Petri Net (HFPN [52]) because it can capture both discrete and continuous behaviors of proteins and other molecules simultaneously in a single simulation model. A simulation model of the OT system was constructed according to the information provided in the KEGG pathway database [31] using the Cell Illustrator software version 3.0 [53] with which the HFPN models can be simulated. Table 3 shows the list of elements (i.e., proteins and other molecules), processes (e.g., activation, suppression, ion transportation), and their parameters (e.g., initial concentration, firing threshold) incorporated into the OT model.

**Table 3:** Elements, processes, and their parameters incorporated into the OT simulation model

Elements/processes	Element abbreviation/process #	Type	Parameter
Odorant	--	Element	Initial value = 10^a^
Olfactory receptor	OR	Element	Initial value = 0 (default)
OR-odorant complex	OR-odorant	Element	Initial value = 0 (default)
G protein olfactory type	G(olf)	Element	Initial value = 0 (default)
Adenylate cyclase	AC	Element	Initial value = 0 (default)
Cyclic adenylic acid	cAMP	Element	Initial value = 0 (default)
Cyclic nucleotide gated channel	CNG	Element	Initial value = 0 (default)
Calcium ion	Ca^2+^	Element	Initial value = 0 (default)
Chloride channel regulator	CLCA	Element	Initial value = 0 (default)
Chloride ion	Cl^-^	Element	Initial value = 0 (default)
Calmodulin	CaM	Element	Initial value = 0 (default)
Phosphodiesterase 1C	PDE1C	Element	Initial value = 0 (default)
Guanylate cyclase activator	GCAP	Element	Initial value = 0 (default)
Guanylate cyclase	pGC	Element	Initial value = 0 (default)
Guanosine 3',5'-cyclic phosphate	cGMP	Element	Initial value = 0 (default)
Protein kinase, cGMP-dependent	PKG	Element	Initial value = 0 (default)
Ca^2+^/CaM-dependent protein kinase	CaMK2	Element	Initial value = 0 (default)
cAMP-dependent protein kinase α	PKA	Element	Initial value = 0 (default)
Phosducin	Phd	Element	Initial value = 0 (default)
Translation	1	Process	Rate = OR*0.05^b^
Degradation	2	Process	Rate = OR*0.05^b^
Binding	3	Process	Rate = Odorant*OR*0.7^c^
Dissociation	4	Process	Rate = Odorant-OR*0.5^c^
Activation	5	Process	Threshold = 2^d^
Activation	6, 8, 10, 14, 15 21, 22, 24, 25, 26	Process	Threshold = 0 (default)
cAMP increase	7	Process	Threshold = 0 (default)
Ion transport	9	Process	Threshold = 0 (default)
Ion transport (depolarization)	11	Process	Threshold = 0 (default)
Degradation	12	Process	Rate = Cl^- ^*0.8^e^
Degradation	13	Process	Rate = 1.0 (default)^f^
Suppression	16, 27, 28	Process	Threshold = 0 (default)
Activation	17	Process	Threshold = 0 (default)^g^
cAMP decomposition	18	Process	Threshold = 0^g ^or 1^g^
Activation	19	Process	Threshold = 0 (default)^h^
cAMP decomposition	20	Process	Threshold = 0^h ^or 1^h^
cGMP increase	23	Process	Threshold = 0 (default)

^a ^Changing this value (5–20) did not affect the results of the simulation.

^b ^Processes 1 and 2 kept the OR at a constant concentration.

^c ^Processes 3 and 4 recovered the OR to its initial concentration after consumption of the odorants.

^d ^Determined *a priori *by test runs. If the threshold was 0, the simulation model did not work and resulted in computation error.

^e ^Process 12 recovered the polarization state of the olfactory receptor neutron model.

^f ^Process 13 was applied in the Ca^2+^-limited model.

^g ^Processes 17 and 18 were applied in the single PDE1C model.

^h ^Processes 19 and 20 were applied in the multiple PDE1C model.

Since the exact parameter settings in modeling biological pathways are generally difficult because of the limited amount of available experimental data [54], we took a simple approach in constructing the OT model. In this approach, almost all parameters in the model were set to the default values (threshold = 0 and no priority) apart from some exceptions explained in the caption of Table 3. After confirming that the odorants successfully elicit depolarization of the olfactory receptor neuron modeled in the OT simulation, we assessed the intensity and longevity of the depolarization signals in the following situations: (*i*) PDE1C circuit is single (single-PDE1C model); (*ii*) the number of PDE1C circuits was increased according to the number of OT-involving PDE1C genes in the stickleback estimated by the RT-PCR-based analysis of co-expression with G(olf) (multiple-PDE1C model); and (*iii*) Ca^2+ ^ion is limited by adding the degradation process of Ca^2+ ^(Ca^2+^-limited model). To further examine the predicted effect of the multiple PDE1C genes on the negative feedback loops and depolarization signals, we performed an *in silico *mutation analysis, where the elements and processes involved in the positive feedback loop were knocked out. The cell system markup language (CSML) files of the OT models constructed and used in the analyses are available online [see Additional file 2].

## Abbreviations

3R-WGD: third-round whole genome duplication; bp: base pairs; CaM: calmodulin; cAMP: cyclic adenylic acid; LSE: lineage-specific expansion; CSML: cell system markup language; CS: conserved synteny; GSP: gene specific primer; HFPN: hybrid functional Petri net; LR-ELW: expected-likelihood weights applied to local rearrangements; LRT: likelihood ratio test; ML: maximum likelihood; OT: olfactory transduction; PDE: phosphodiesterase; RT-PCR: reverse transcription polymerase chain reaction.

## Authors' contributions

YS, YH, and MN designed the study. YH collected stickleback fish samples and prepared the tissue samples for molecular work. YS carried out the molecular work and analyses, and drafted the manuscript. MN participated in coordination and helped to draft the manuscript. All authors read and approved the final version of the manuscript.

## Supplementary Material

Additional File 1 **Supplementary figure and tables**. This PDF file includes supplementary figures S1–S2 and tables S1–S2. Click here for file

Additional File 2 **CSML files of the simulation models**. This ZIP file includes CSML files of the simulation models constructed and used in this study. Click here for file
