# Supplementary material for: Evolution of multiple phosphodiesterase isoforms in stickleback involved in cAMP signal transduction pathway
Source: BMC Syst Biol. 2009 Feb 20;3:23. doi: 10.1186/1752-0509-3-23 (PMC2653465; doi:10.1186/1752-0509-3-23)
Supplement: Additional File 1 — Supplementary figure and tables. This PDF file includes supplementary figures S1–S2 and tables S1–S2. [file 1752-0509-3-23-S1.pdf]

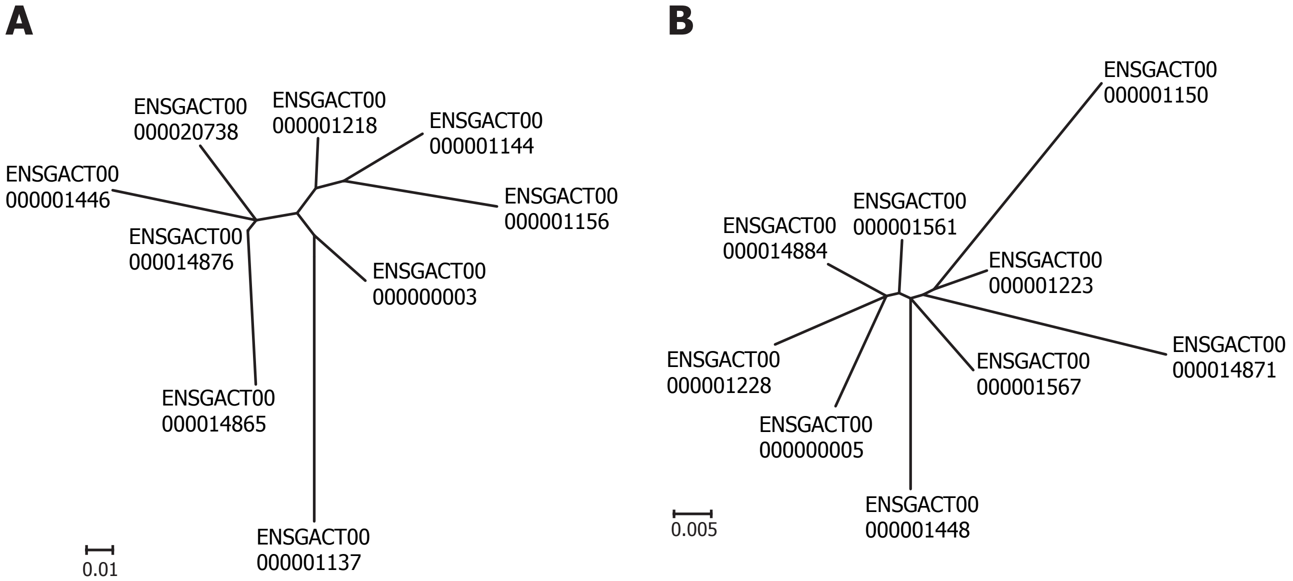

Figure S1. Maximum-likelihood trees of two families of unknown genes located around the PDE1Cb loci in stickleback. One of the families was analyzed using 207 base pairs (bp) of the alignable region of protein-coding sequence based on TrN + Γ model of nucleotide substitution (panel **A**), and the other family was analyzed using 567 bp of the alignable region of protein-coding sequence based on HKY + Γ model of nucleotide substitution (panel **B**).

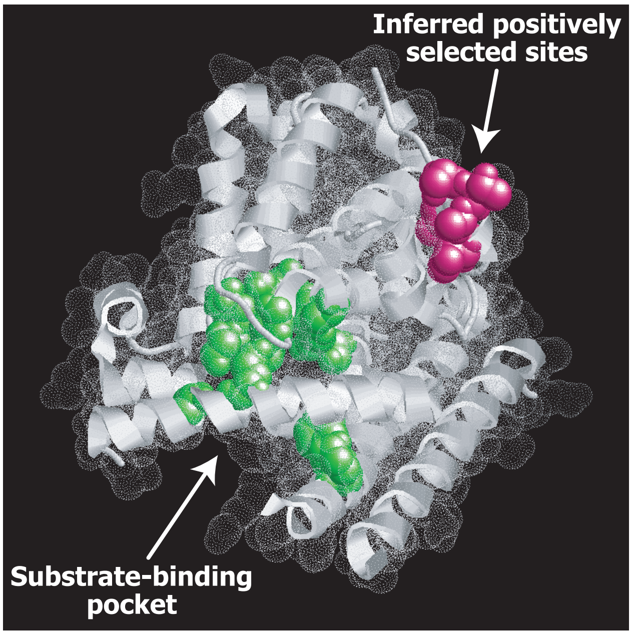

Figure S2. Distributions of locations of inferred positively-selected amino acid sites in the evolution of stickleback PDE1Cbs (colored in magenta) and amino acid sites that are involved in a substrate-binding pocket of phosphodiesterase 1 enzymes (colored in green). The three-dimensional graphical model was constructed using the program RasMol. Human phosphodiesterase 1B (PDB ID: 1TAZ) was used as a reference structure based on a query to the homology modeling server SWISS-MODEL using amino acid sequences of stickleback PDE1Cb.

Table S1 - List of genes located on the conserved syntenies around the vertebrate PDE locus (loci)

Species	Chromosome/ Scaffold #	Name	Ensembl ID	Location
Human	Chr. 7	GHRHR	ENSP00000320180	30,970,161-30,990,114
		ADCYAP1R1	ENSP00000306620	31,058,650-31,117,614
		NEUROD6	ENSP00000297142	31,343,607-31,347,063
		CCDC129	ENSP00000313062	31,523,503-31,659,828
		C7orf16	ENSP00000340125	31,693,372-31,714,593
		**PDE1C**	**ENSP00000284920**	**31,757,318-32,305,466**
		LSM5	ENSP00000223084	32,492,944-32,496,502
		KIAA0241	ENSP00000315568	32,501,701-33,045,041
		KBTBD2	ENSP00000302586	32,874,311-32,897,897
		AC018648.5	ENSP00000335479	32,922,952-32,949,307
	Chr. 1	ABCD3	ENSP00000326880	94,656,599-94,756,670
		PTBP2	ENSP00000236228	96,959,927-97,052,937
		PPAPR5	ENSP00000263177	99,128,391-99,243,037
		RP4-788L13.1	ENSP00000263178	99,502,436-99,547,734
	Chr. 10	RSU1	ENSP00000339521	16,672,625-16,899,468
		TRDMT1	ENSP00000324263	17,224,988-17,283,687
Chicken	Chr. 2	NP_001012876 .1	ENSGALP00000019958	48,770,436-48,992,328
		NEUROD6	ENSGALP00000019951	48,697,032-48,698,045
		C7orf16	ENSGALP00000019940	48,546,048-48,556,659
		No description	ENSGALP00000030477	48,522,694-48,522,901
		**PDE1C**	**ENSGALP00000019935**	**48,463,226-48,584,380**
		No description	ENSGALP00000019936	48,290,077-48,295,790
		LSM5	ENSGALP00000019908	48,174,060-48,177,770
		KIAA0241	ENSGALP00000019901	48,135,972-48,171,609
		KBTBD2	ENSGALP00000019897	48,122,874-48,127,240
Xenopus	Sca. 588	No description	ENSXETP00000050587	2,632-14,833
		ITGA9	ENSXETP00000050595	27,370-264,562
		NEUROD6	ENSXETP00000050601	393,890-394,903
		C7orf16	ENSXETP00000050625	530,823-534,682
		**PDE1C**	**ENSXETP00000050616**	**527,660-614,294**
		No description	ENSXETP00000022786	652,885-654,078

Zebrafish	Zv7_sca. 2640	No description	ENSDARESTP00000035364	34,492-39,056
		zgc:73275	ENSDARP00000065350	158,849-178,673
		No description	ENSDARESTP00000008040	174,807-178,673
		No description	ENSDARESTP00000008042	215,539-316,483
		**PDE1Ca**	**ENSDARP00000067654**	**300,136-331,748**
Medaka	Chr. 17	No description	ENSORLP00000022159	29,666,544-29,667,524
		No description	ENSORLP00000022227	30,150,667-30,181,732
		ADCYAP1R1	ENSORLP00000022250	30,187,008-30,206,172
		FYCO1	ENSORLP00000022262	30,259,354-30,281,847
		XCR1	ENSORLP00000022264	30,285,683-30,286,675
		NEUROD6	ENSORLP00000022266	30,354,947-30,355,951
		**PDE1Ca**	**ENSORLP00000022278**	**30,378,326-30,409,681**
		No description	ENSORLP00000022280	30,445,574-30,447,865
		No description	ENSORLP00000022291	30,533,140-30,539,249
		No description	ENSORLP00000022292	30,551,838-30,559,225
		No description	ENSORLP00000022301	30,562,554-30,577,469
		RGS20	ENSORLP00000022303	30,584,840-30,590,255
Medaka	Chr. 4	ADCYAP1R1	ENSORLP00000016315	23,140,165-23,151,683
		FYCO1	ENSORLP00000020832	31,574,629-31,594,664
		XCR1	ENSORLP00000020837	31,597,704-31,601,230
		ATF6	ENSORLP00000020861	31,630,439-31,659,486
Medaka	Chr. 20	C14orf122	ENSORLP00000016704	22,257,445-22,262,989
		IRF9	ENSORLP00000016713	22,263,930-22,267,315
		RNF31	ENSORLP00000016757	22,269,021-22,282,027
		PSME2	ENSORLP00000016814	22,284,737-22,287,783
		LSM5	ENSORLP00000016836	22,287,820-22,289,086
		KIAA0241	ENSORLP00000016864	22,291,176-22,297,892
		KBTBD2	ENSORLP00000016874	22,299,997-22,303,357
Tetraodon	Un_random	No description	ENSTNIT00000022040	20,600,675-20,614,560
		ADCYAP1R1	ENSTNIT00000022041	20,618,267-20,626,114
		FYCO1	ENSTNIT00000022042	20,645,306-20,652,836
		No description	ENSTNIT00000022043	20,661,851-20,663,214
		NEUROD6	ENSTNIT00000022044	20,680,898-20,681,908
		No description	ENSTNIT00000022045	20,691,942-20,693,382
		**PDE1Ca**	**ENSTNIT00000022046**	**20,697,673-20,711,039**
		No description	ENSTNIT00000022047	20,754,673-20,759,204
		No description	ENSTNIT00000022048	20,768,737-20,770,044
		No description	ENSTNIT00000022049	20,772,346-20,775,272

		RGS20	ENSTNIT00000022050	20,779,427-20,780,481
Tetraodon	Un_random	No description	ENSTNIT00000006561	9,853,352-9,856,797
		No description	ENSTNIT00000002026	9,869,857-9,873,483
		PPAPR5	ENSTNIT00000008124	9,976,996-9,981,214
		No description	ENSTNIT00000008123	9,986,453-9,992,564
		ABCD3	ENSTNIT00000008122	9,995,328-10,006,686
		**PDE1Cb**	**ENSTNIT00000008121**	**10,018,802-10,025,299**
		No description	ENSTNIT00000008120	10,025,737-10,027,797
		TRDMT1	ENSTNIT00000003137	10,032,770-10,037,838
		RSU1	ENSTNIT00000008119	10,028,342-10,031,828
		No description	ENSTNIT00000006614	10,050,628-10,054,476
		PTBP2	ENSTNIT00000022375	10,078,957-10,086,773
		TGFBR1	ENSTNIT00000022376	10,155,406-10,160,175
Tetraodon	Chr. 1	No description	ENSTNIT00000022967	175,149-177,022
		FYCO1	ENSTNIT00000009582	14,265,310-14,274,311
		ATF6	ENSTNIT00000013791	14,699,758-14,711,482
		ADCYAP1R1	ENSTNIT00000009160	22,911,954-22,915,993
Tetraodon	Un_random	No description	ENSTNIT00000011723	89,543,441-89,545,958
		KBTBD2	ENSTNIT00000011724	89,547,867-89,551,070
		KIAA0241	ENSTNIT00000011725	89,553,567-89,559,790
		LSM5	ENSTNIT00000011726	89,560,445-89,561,959
		PSME2	ENSTNIT00000011727	89,561,973-89,563,735
		RNF31	ENSTNIT00000002130	89,564,786-89,570,932
		IRF9	ENSTNIT00000011730	89,571,760-89,573,470
Stickleback	GroupIII	No description	ENSGACT00000022685	12,992,509-12,997,481
		No description	ENSGACT00000022693	13,000,537-13,002,163
		No description	ENSGACT00000022721	13,012,101-13,017,190
		**PDE1Ca**	**ENSGACP00000022683**	**13,038,856-13,072,774**
		NEUROD6	ENSGACT00000022727	13,086,480-13,087,485
		XCR1	ENSGACT00000022730	13,102,288-13,103,869
		FYCO1	ENSGACT00000022735	13,106,071-13,116,555
		ADCYAP1R1	ENSGACT00000022739	13,136,933-13,151,610
		No description	ENSGACT00000022744	13,156,738-13,171,395
		EEF1D	ENSGACT00000022748	13,174,470-13,181,073
Stickleback	GroupVIII	ADCYAP1R1	ENSGACT00000007161	4,746,933-4,754,805
		ATF6	ENSGACT00000015511	15,070,643-15,090,855
		XCR1	ENSGACT00000015563	15,115,220-15,116,795

		FYCO1	ENSGACT00000015578	15,118,216-15,127,957
Stickleback	GroupXXI	No description	ENSGACT00000007318	11,434,878-11,481,114
		No description	ENSGACT00000007321	11,470,556-11,471,458
		LSM5	ENSGACT00000007338	11,494,158-11,496,788
		KIAA0241	ENSGACT00000007357	11,497,735-11,506,320
		KBTBD2	ENSGACT00000007365	11,510,842-11,514,721
		No description	ENSGACT00000007368	11,523,776-11,524,908
Stickleback	Sca. 154	**PDE1Cb1**	ENSGACT00000000002	1,880-13,338
		No description	ENSGACT00000000003	47,616-48,140
		No description	ENSGACT00000000004	56,205-56,936
		No description	ENSGACT00000000005	171,475-172,632
Stickleback	Sca. 37	No description	ENSGACT00000001124	484,382-495,230
		No description	ENSGACT00000001129	498,939-502,916
		No description	ENSGACT00000001132	508,232-543,386
		No description	ENSGACT00000001137	552,712-648,114
		**PDE1Cb2**	**ENSGACT00000001141**	**629,537-638,040**
		No description	ENSGACT00000001144	686,847-687,371
		No description	ENSGACT00000001150	731,296-732,371
		No description	ENSGACT00000001156	746,347-746,871
		HECW1	ENSGACT00000001165	759,377-785,300
		No description	ENSGACT00000001168	790,394-802,495
Stickleback	Sca. 134	No description	ENSGACT00000001216	68,828-95,132
		No description	ENSGACT00000001218	116,764-117,285
		No description	ENSGACT00000001220	131,619-133,256
		No description	ENSGACT00000001223	147,433-148,553
		No description	ENSGACT00000001225	158,036-225,098
		No description	ENSGACT00000001228	217,772-218,859
		**PDE1Cb3**	**ENSGACP00000001232**	**230,028-237,051**
Stickleback	Sca. 223	No description	ENSGACT00000001439	48,196-50,808
		**PDE1Cb4**	**ENSGACP00000001443**	**61,833-69,270**
		No description	ENSGACT00000001446	73,738-74,265
		No description	ENSGACT00000001448	79,087-80,059
Stickleback	Sca. 215	No description	ENSGACT00000001561	39,095-40,194
		**PDE1Cb5**	**ENSGACP00000001564**	**54,335-64,086**
		No description	ENSGACT00000001567	72,788-73,760

Stickleback	Sca. 94	No description	ENSGACT00000014862	340,146-340,661
		No description	ENSGACT00000014865	351,658-352,197
		No description	ENSGACT00000014868	351,830-387,624
		No description	ENSGACT00000014871	381,420-382,212
		No description	ENSGACT00000014873	407,290-409,612
		No description	ENSGACT00000014876	414,836-415,357
		**PDE1Cb6**	**ENSGACP00000014854**	**461,258-473,783**
		No description	ENSGACT00000014884	483,064-484,036
Stickleback	Sca. 188	No description	ENSGACT00000020738	54,230-54,751
		No description	ENSGACT00000020739	62,923-64,267
		**PDE1Cb7**	**ENSGACP00000020708**	**77,715-86,372**
Stickleback	Sca. 809	**PDE1Cbx**	**ENSGACP00000001336**	**3,388-7,099**

Table S2 - List of chordate species and PDE1 genes analyzed in this study with Ensembl Gene IDs

Species	Scientific name	Name	Ensembl ID
Human	*Homo sapiens*	PDE1C	ENSP00000284920
		PDE1A	ENSP00000329112
Chicken	*Gallus gallus*	PDE1C	ENSGALP00000019935
		PDE1A	ENSGALP00000014423
Frog	*Xenopus tropicalis*	PDE1C	ENSXETP00000050616
		PDE1A [a]	--
Pufferfish	*Tetraodon nigroviridis*	PDE1Ca	GSTENP00034501001 [b]
		PDE1Cb	GSTENP00009446001 [c]
		PDE1A [a]	--
Medaka	*Oryzias latipes*	PDE1Ca	ENSORLP00000022278
		PDE1A	ENSORLP00000017334
Stickleback	*Gasterosteus aculeatus*	PDE1Ca	ENSGACP00000022683
		PDE1Cb1	ENSGACP00000000002
		PDE1Cb2	ENSGACP00000001141
		PDE1Cb3	ENSGACP00000001232 [d]
		PDE1Cb4	ENSGACP00000001443 [d]
		PDE1Cb5	ENSGACP00000001564
		PDE1Cb6	ENSGACP00000014854
		PDE1Cb7	ENSGACP00000020708
		PDE1Cx	ENSGACP00000001336 [e]
		PDE1A	ENSGACP00000010793
Zebrafish	*Danio rerio*	PDE1Ca	ENSDARP00000067654 [e]
		PDE1A	ENSDARP00000080640
Ascidian	*Ciona intestinalis*	PDE1	ENSCINP00000013646

[a] A correponding genes was not found by BLAST searches against the genome sequence.

[b] Current Ensembl ID is ENSTNIT00000022046.

[c] Current Ensembl ID is ENSTNIT00000008121.

[d] Full length sequences were predicted using the program Wise2.

[e] Only a partial sequence was found.
